# The Effect of Protein-Rich Extract from *Bombyx Batryticatus* against Glutamate-Damaged PC12 Cells Via Regulating γ-Aminobutyric Acid Signaling Pathway

**DOI:** 10.3390/molecules25030553

**Published:** 2020-01-28

**Authors:** Li-Ying He, Mei-Bian Hu, Ruo-Lan Li, Rong Zhao, Lin-Hong Fan, Li Wang, Wei Peng, Yu-Jie Liu, Chun-Jie Wu

**Affiliations:** 1 College of Pharmacy, Chengdu University of Traditional Chinese Medicine, Chengdu 611137, China; heliying@stu.cdutcm.edu.cn (L.-Y.H.); hmbcdtcm@163.com (M.-B.H.); lee69205@163.com (R.-L.L.); 15228279571@163.com (R.Z.); fanlinhong1996@163.com (L.-H.F.); liwang201812@163.com (L.W.); pengwei002@126.com (W.P.); 2 School of Pharmacy, Chengdu Medical College, Chengdu 610500, China

**Keywords:** protein from *Bombyx batryticatus*, epilepsy, glutamate, PC12 cells, protective effects, γ-aminobutyric acid

## Abstract

*Bombyx Batryticatus* (BB) is a known traditional Chinese medicine (TCM) utilized to treat convulsions, epilepsy, cough, asthma, headaches, etc. in China for thousands of years. This study is aimed at investigating optimum extraction of protein-rich extracts from BB (BBPs) using response surface methodology (RSM) and exploring the protective effects of BBPs against nerve growth factor (NGF)-induced PC12 cells injured by glutamate (Glu) and their underlying mechanisms. The results indicated optimum process of extraction was as follows: extraction time 1.00 h, ratio of liquid to the raw material 3.80 mL/g and ultrasonic power 230.0 W. The cell viability of PC12 cells stimulated by Glu was determined by CCK-8 assay. The levels of γ-aminobutyric (GABA), interleukin-1β (IL-1β), interleukin-4 (IL-4), tumor necrosis factor-α (TNF-α), 5-hydroxytryptamine (5-HT) and glucocorticoid receptor alpha (GR) in PC12 cells were assayed by ELISA. Furthermore, the Ca^2+^ levels in PC12 cells were determined by flow cytometry analysis. Protein and mRNA expressions of GABAA-Rα1, NMDAR1, GAD 65, GAD 67, GAT 1 and GAT 3 in PC12 cells were evaluated by real-time polymerase chain reaction (RT-PCR) and Western blotting assays. Results revealed that BBPs decreased toxic effects due to Glu treatment and decreased Ca^2+^ levels in PC12 cells. After BBPs treatments, levels of GABA and 5-HT were increased and contents of TNF-α, IL-4 and IL-1β were decreased in NGF-induced PC12 cells injured by Glu. Moreover, BBPs up-regulated the expressions of GABAA-Rα1, GAD 65 and GAD 67, whereas down-regulated that of NMDAR1 GAT 1 and GAT 3. These findings suggested that BBPs possessed protective effects on NGF-induced PC12 cells injured by Glu via γ-Aminobutyric Acid (GABA) signaling pathways, which demonstrated that BBPs has potential anti-epileptic effect in vitro. These findings may be useful in the development of novel medicine for the treatment of epilepsy.

## 1. Introduction

Epilepsy, which is characterized by recurrent unprovoked seizures, is a common neurological disorder [1,2]. Antiepileptic drugs (AEDs) still remain the mainstay for the treatment of epilepsy but these drugs have many side effects, such as drowsiness, impaired memory and blurred vision, etc [3]. The occurrence of epilepsy is not only related to the imbalance between excitatory neurotransmitters and inhibitory neurotransmitters, but also related to the abnormal ion channel function (mainly Ca^2+^) caused by the abnormal function of glial cells, the release of inflammatory factors, and the release of glucocorticoids [4]. However, the imbalance between glutamate (Glu) and γ-aminobutyric acid (GABA) is considered as a main factor of the occurrence of epilepsy [5]. GABA is an important inhibitory neurotransmission, and decrease of its levels is regarded as one of the important epilepsy pathogenesis [6]. GABA signaling pathways mainly include GABA receptors, glutamic acid decarboxylase (GADs) and GABA-transporters (GATs). After a GABA receptor is activated, the chloride channel is opened, and a large number of chloride ions flow in rapidly, which leads to the hyperpolarization of postsynaptic membrane, thus inhibiting the over discharge and postsynaptic facilitation of regulatory neurons and playing a postsynaptic inhibitory role. GABA receptors can be divided into GABAA, GABAB and GABAC, among which GABAA is most closely related to epilepsy [7,8,9,10,11]. GADs, as an enzyme, could promote glutamic acid to synthesize neuronal GABA, which is encoded by two different genes, GAD2 (GAD 65) and GAD1 (GAD 67) [10]. GAD 65 is mainly present in the nerve terminal, whereas GAD 67 is diffusely distributed in the cell body as well as nerve terminals [8,9]. GAD 67 plays the major role for GABA production in the embryonic brain, whereas the contribution of GAD 65 begins to increase after birth. GAT 1 and GAT 3, as GATs, are expressed in nerve terminals and glia to remove GABA from the synaptic cleft in the brain, and lack of expression of GATs would enhance the ability of GABA to spread through the extracellular space [12]. Thus, the GABA signaling pathway was generally selected to explore mechanism of antiepileptic action.

In addition, inflammatory response is also a common pathological mechanism of epilepsy and may reflect the severity of the disease to some extent [13]. It is reported that inflammatory cytokines, such as IL-1β, IL-4 and TNF-α are important regulators of human inflammatory response, whose imbalance and overproduction can lead to neuronal degeneration and induce seizures [14]. The content levels of IL-1β, IL-4 and TNF-α are rapidly increased when brain is subjected to acute insults like seizures [15,16]. Studies have shown that repeated seizures can induce immune inflammatory via activating the IL-1β signaling pathway in the hippocampus [17]. TNF-α treated rats showed more prolonged epileptic form discharges than control rats [18]. It is reported that IL-4 can increase microglial activation [19]. In addition, some studies have confirmed that 5-HT can significantly inhibit epilepsy [13]. Glucocorticoids possess many regulating functions on the nervous system through the glucocorticoid receptor (GR), such as regulating the levels of neurotransmitters, signal transduction and neuronal differentiation, etc [20].

PC12 cells (rat pheochromocytoma cells) with good neuronal properties is commonly considered to be a useful in vitro model of researches on neurological diseases, such as stroke, senile dementia (SD), epilepsy and other diseases [21,22]. NGF (nerve growth factor) is substances that promote neuronal survival, differentiation, and regeneration [23,24]. The model of PC12 neuron-like cells stimulated by NGF is commonly used for studying the differentiation of nerve cells. In addition, Glu is the primary excitatory neurotransmitter in the brain as well as the immediate precursor of the inhibitory GABA, which acts on its postsynaptic receptors to mediate excitatory neurotransmission, and it is involved in neural development and synaptic plasticity [25]. High Glu concentrations can lead to neurodegenerative diseases. An indirect marker of Glu toxicity is Ca^2+^ influx [26,27]. Moreover, the increase of Glu was positively correlated with the intensity of epileptic activity [28]. NMDAR1, as a receptor of Glu, is closely related to epilepsy, and over activation of NMDAR receptor is considered as one of epilepsy pathogenesis [29]. Therefore, the model of NGF-induced PC12 cell injured by Glu was generally selected to study neurological disorders in vitro, such as Alzheimer’s disease and epilepsy [30,31].

*Bombyx batryticatus* (BB) is the dried larva of *Bombyx mori L*. (silkworm of 4–5 instars) infected by *Beauveria bassiana* (Bals.) Vuill [32]. It is reported that BB possess significant anticonvulsant and antiepileptic, anticoagulant, antitumor, antibacterial and antifungal effects and other effects [33], and it has been used to treat convulsion, epilepsy, headache, migraine, facial paralysis, hemiplegia, cough, asthma, as well as other diseases [34,35]. In addition, treatments of convulsions and epilepsy are the main traditional applications of BB, and a large number of researches have shown that extracts/compounds isolated from BB possess significant anticonvulsant and antiepileptic effects on different animal models [34,36]. A recent study has shown that extracts from BB reduce the neurotoxic effects of Glu agonists n-methyl-d-aspartic acid (NMDA) and kainic acid on the hippocampus [37]. Current investigations indicated that BB contains multiple compounds, including protein and peptides, fatty acids, flavonoids, steroids, polysaccharide and others [38]. As an important animal traditional Chinese medicine, protein components are the one of the main components in BB [39]. Protein components in BB has a protective effect against amyloid-β (Aβ)-induced cytotoxicity in astrocyte cells through the inhibition of lipid peroxidation and protection of anti-oxidative enzymes [37], and protective effects on H_2_O_2_-induced oxidative stress in PC12 cells via PI3K/Akt signaling pathways [35]. Therefore, it can be found that “protective” molecules can exhibit different activities depending on the study model chosen [40,41]. However, the protective effect and mechanism of protein components in BB (BBPs) on Glu-induced PC12 cells has not been reported currently. Therefore, the aim of the present study is to study the effect of BBPs on NGF-induced PC12 cells injured by Glu and explore the underlying mechanism. The present study will provide scientific basis for BB of traditional usage in treating convulsions and epilepsy.

## 2. Results

### 2.1. Optimization of Extraction Conditions

The effect of different extraction times (0.25, 0.5, 0.75, 1 and 1.25 h), ratio of water to raw material (2, 3, 4, 5 and 6 mL/g) and ultrasonic power (140, 180, 220, 260, and 300 W) on the yield of BBPs were investigated in our previous study. According to the survey results, 0.75–1.25 h, 3–5 mL/g and 180–260 W were selected as extraction time, ratio of water to raw material and ultrasonic power for RSM study.

The experimental data were analyzed by multiple regression, the predicted response Y for extraction yield of AUP (%) can be obtained by the following second-order polynomial equation: Y = 2.49 + 0.070 A − 0.094 B + 0.27 C − 0.19 AB + 1 × 10^−2^ AC + 0.032 BC − 0.39 A^2^ − 0.29 B^2^ − 0.38 C^2^. The results of the analysis of goodness-of-fit, variance, and the adequacy of the mode is shown in Table 1. According to the response surface results, it can be concluded that optimum condition for yield of AUP was obtained: extraction time 1.04 h, ratio of solid-liquid 3.81 mL/g and ultrasonic power 234.03 W.

### 2.2. Effects of BBPs and Glu on Cell Viability of PC12 Cells

**Figure 1:**
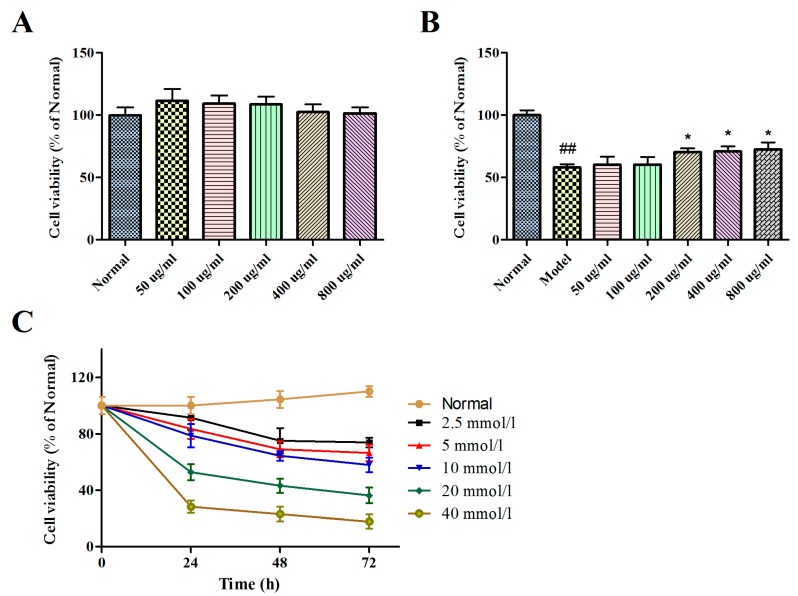
Protective effects of BBPs on the cell viability of NGF-induced PC12 cells injured by Glu. (**A**) The effect of BBPs at different concentrations on the cell viabilities of NGF-induced PC12 cells. (**B**) The effects of BBPs on the cell viability of NGF-induced PC12 cells stimulated by Glu. (**C**) The effects of Glu at different concentrations on the cell viabilities of NGF-induced PC12 cells. PC12 cells (induced by 50 ng/mL NGF for 48 h) were treated with BBPs at concentrations of 200, 400 and 800 μg/mL for 24 h, subsequently subjected to Glu at the concentration of 20 mmol/L for a further 24 h. Normal group: NGF-induced cells without BBPs and Glu; Model group: NGF-induced cells without BBPs, but with 20 mmol/L Glu; BBPs: protein extracts in *Bombyx batryticatus.* The values represent mean ± SD (*n* = 3). ^##^ *P* < 0.01, vs. normal group; * *P* < 0.05, vs. model group.

The effect results of BBPs and Glu on the cell viabilities of NGF-induced PC12 cells were presented in Figure 1A,C. It could be found that BBPs did not show any toxicity of up to the concentration of 800 μg/mL in cells. However, Glu at concentrations of 2.5–40 mmol/L has significantly toxicity on normal PC12 cells under different culture time (0, 24, 48 and 72 h), and at concentrations of 20 mmol/L for 24 h, the cell viability of Glu on NGF-induced PC12 cells was 52.75%. Thus, Glu at concentrations of 20 mmol/L for 24 h was used to establish injure induced by Glu.

The effects of BBPs on the cell viability of NGF-induced PC12 cells injured by Glu were evaluated by CCK-8 assay. As shown in Figure 1B, the cell viability was significantly reduced by treatment with Glu (*P* < 0.01). However, BBPs decreased the toxic effects due to Glu treatment from the concentrations of 200 to 800 μg/mL, compared to the model cells (*P* < 0.05). Consequently, the results indicated that BBPs had a potential protective effect against the Glu-induced injury in PC12 cells.

### 2.3. Effects of BBPs on GABA, IL-1β, IL-4, 5-HT, TNF-α and GRα in NGF-Induced PC12 Cells Injured by Glu

**Figure 2:**
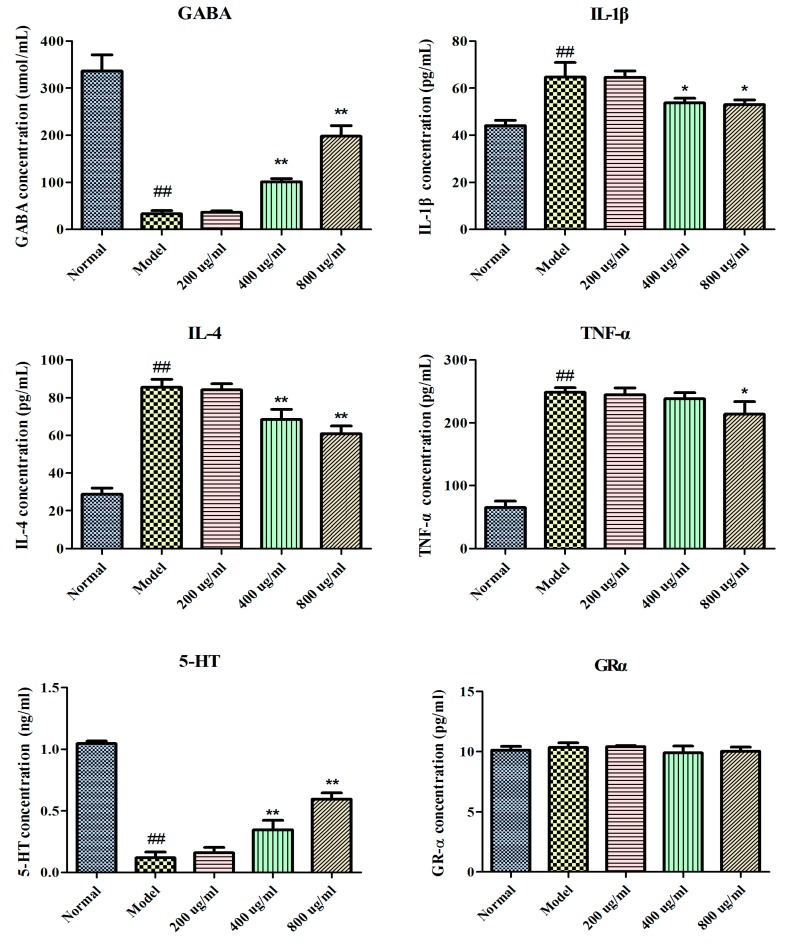
Effects of BBPs on GABA, IL-1β, IL-4, TNF-α, 5-HT and GRα in NGF-induced PC12 cells injured by Glu. The levels of GABA, IL-1β, IL-4, TNF-α and GRα were determined by corresponding ELISA kits. PC12 cells (induced by 50 ng/mL NGF for 48 h) were treated with BBPs at concentrations of 200, 400 and 800 μg/mL for 24 h, subsequently subjected to Glu at the concentration of 20 mmol/L for a further 24 h. Normal group: NGF-induced cells without BBPs and Glu; Model group: NGF-induced cells without BBPs, but with 20 mmol/L Glu; BBPs: protein extracts in *Bombyx batryticatus* The values represent mean ± SD (*n* = 6). ^##^ *P* < 0.01, vs. normal group; * *P* < 0.05 and ** *P* < 0.01, vs. model group.

The effects of BBPs on the levels of GABA, IL-1β, IL-4, TNF-α, 5-HT and GRα were detected in NGF-induced PC12 cells injured by Glu. As shown in Figure 2, the levels of GABA and 5-HT were significantly decreased (*P* < 0.01), whereas the TNF-α, IL-4 and IL-1β contents were increased (*P* < 0.01) in model cells compared with normal cells. Interestingly, BBPs (400 and 800 μg/mL) significantly reduced IL-1β and IL-4 contents compared with the model cells (*P* < 0 05). The GABA and 5-HT level was obviously increased in BBP-treated cells 400 and 800 μg/mL (*P* < 0 01), compared with the model cells. In addition, BBPs showed no any effect on GRα level. BBPs (200 ug/mL) on GABA, IL-1Β, IL-4, 5-HT, TNF-α and GRα in Glu-damaged PC12 cells showed no marked difference compere with the model cells.

### 2.4. Effects of BBPs on Ca^2+^ Levels of NGF-Induced PC12 Cells Injured by Glu

**Figure 3:**
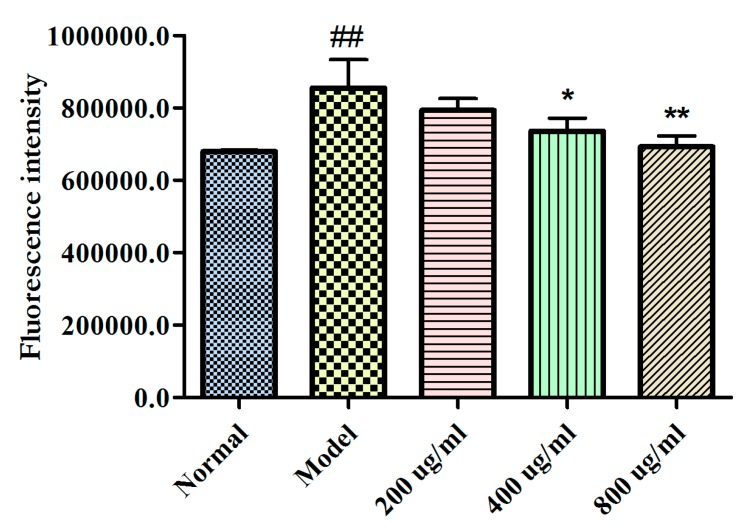
Effects of BBPs on Ca^2+^ levels of NGF-induced PC12 cells injured by Glu. The intracellular Ca^2+^ level by the flow cytometry (FCM) assay. PC12 cells (induced by 50 ng/mL NGF for 48 h) were treated with BBPs at concentrations of 200, 400 and 800 μg/mL for 24 h, subsequently subjected to Glu at the concentration of 20 mmol/L for a further 24 h. Normal group: NGF-induced cells without BBPs and Glu; Model group: NGF-induced cells without BBPs, but with 20 mmol/L Glu; BBPs: protein extracts in *Bombyx batryticatus.* The values represent mean ± SD (*n* = 6). ^##^ *P* < 0.01, vs. normal group; * *P* < 0.05 and ** *P* < 0.01, vs. model group.

As shown in Figure 3, the Ca^2+^ level of the model cells significantly increased compared with that of normal cells (*P* < 0.01). However, BBPs (400, and 800 μg/mL) could dose-dependently reduce the Ca^2+^ level in PC12 cells injured by Glu (*P* < 0.05, and *P* < 0.01), compared with that in the model cells.

### 2.5. Effects of BBPs on mRNA Expressions of GABAA-Rα1, NMDAR1, GAD 65, GAT 1, GAT 3 and GAD 67 in NGF-Induced PC12 Cells Injured by Glu

**Figure 4:**
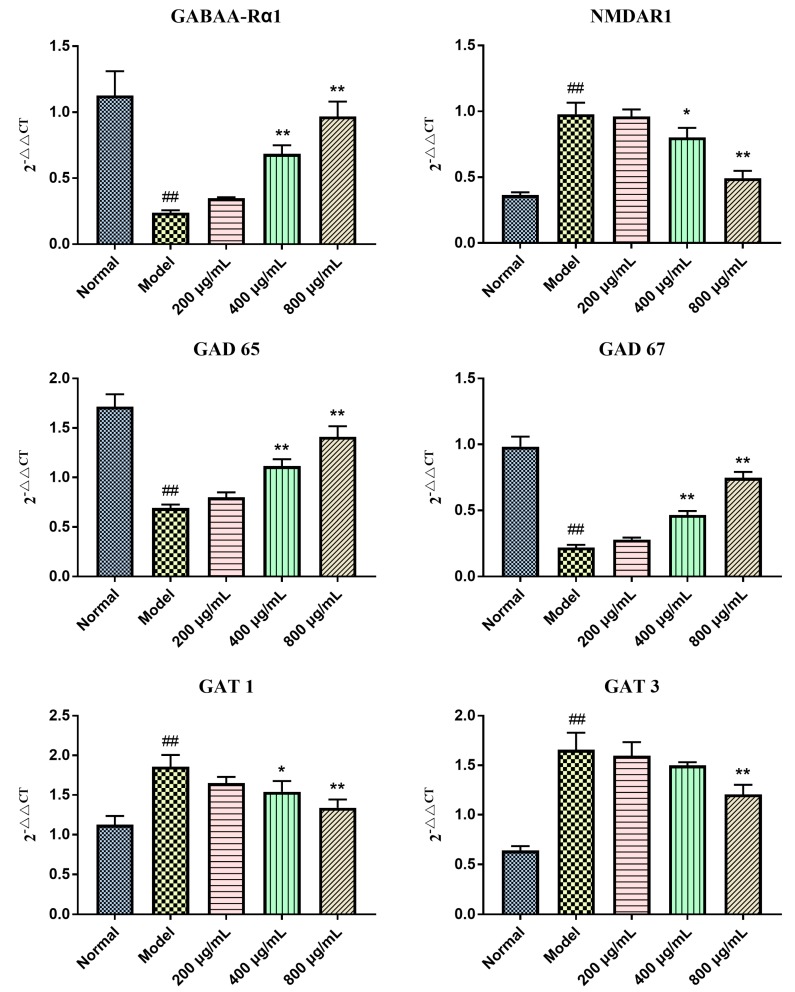
Effects of BBPs on mRNA expressions of *GABAA-Rα1*, *GAD 65, GAD 67, NMDAR1*, *GAT 1* and *GAT 3* in NGF-induced PC12 cells injured by Glu. PC12 cells (induced by 50 ng/mL NGF for 48 h) were treated with BBPs at concentrations of 200, 400 and 800 μg/mL for 24 h, subsequently subjected to Glu at the concentration of 20 mmol/L for a further 24 h. GABAA-Rα1, NMDAR1, GAD65, GAT 1, GAT 3 and GAD67 mRNA were detected by real-time PCR and β-actin was detected as the control. Normal group: NGF-induced cells without BBPs and Glu; Model group: NGF-induced cells without BBPs, but with 20 mmol/L Glu; BBPs: protein extracts in *Bombyx batryticatus.* The values represent mean ± SD (*n* = 3). ^##^ *P* < 0.01, vs. normal group; * *P* < 0.05 and ** *P* < 0.01, vs. model group.

To explore the protective mechanism of BBPs on NGF-induced PC12 cells injured by Glu, mRNA expressions of GABAA-Rα1, NMDAR1, GAD 65, GAT 1, GAT 3 and GAD 67 were detected in the present study. As shown in Figure 4, the mRNA expressions of *NMDAR1, GAT 1*, and *GAT 3* was upregulated, whereas expressions of GABAA-Rα1, GAD 65 and GAD 67 in the model cells were obviously down regulated, compared with that of normal cells (*P* < 0.01). BBPs significantly increased the mRNA expressions of GABAA-Rα1, GAD65 and GAD67 at the concentrations of 400 and 800 μg/mL (*P* < 0.01, *P* < 0.01) and decreased GAT 1 (*P* < 0.05, *P* < 0.01) and NMDAR1(*P* < 0.05, *P* < 0.01) expression in NGF-induced PC12 cells injured by Glu relative to the model cells. Only BBPs at a concentration of 800 μg/mL obviously decreased *GAT 3* (*P* < 0.01) expression.

### 2.6. Effects of BBPs on Protein Expressions of GABAA-Rα1, NMDAR1, GAD 65, GAT 1, GAT 3 and GAD 67 in NGF-Induced PC12 Cells Injured by Glu

**Figure 5:**
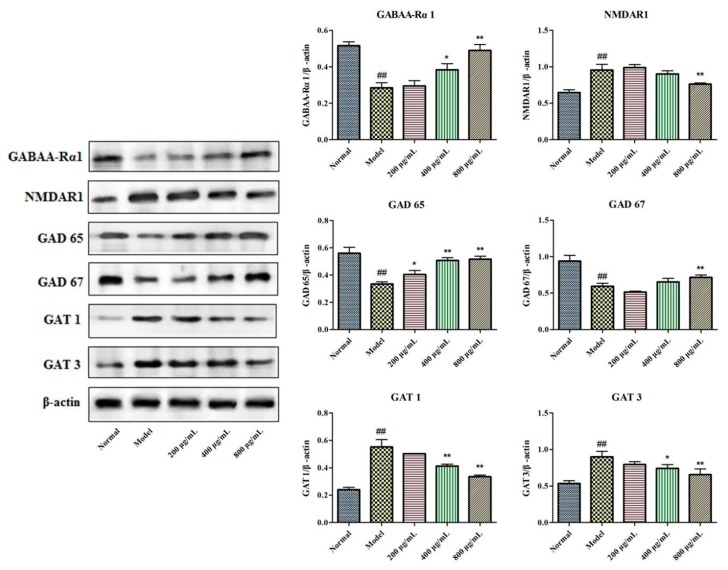
Effects of BBPs on protein expressions of GABAA-Rα1, NMDAR1, GAD65, GAT-1, GAT-3 and GAD67 in NGF-induced PC12 cells injured by Glu. PC12 cells (induced by 50 ng/mL NGF for 48 h) were treated with BBPs at concentrations of 200, 400 and 800 μg/mL for 24 h, subsequently subjected to Glu at the concentration of 20 mmol/L for a further 24 h. GABAA-Rα1, NMDAR1, GAD65, GAT-1, GAT-3 and GAD67 proteins were detected by Western blotting, whereas β-actin was detected as the control. Normal group: NGF-induced cells without BBPs and Glu; Model group: NGF-induced cells without BBPs, but with 20 mmol/L Glu; BBPs: protein extracts in *Bombyx batryticatus.* The values represent mean ± SD (*n* = 3). ^##^ *P* < 0.01, vs. normal group; * *P* < 0.05 and ** *P* < 0.01, vs. model group.

To explore the protective mechanism of NGF-induced PC12 cells injured by Glu, protein expressions of GABAA-Rα1, NMDAR1, GAD 65, GAT 1, GAT 3 and GAD 67 were detected in the present study. It could be found in Figure 5 that protein expressions of GABAA-Rα1, GAD 65 and GAD 67 were significantly decreased in model cells induced by Glu, whereas expression level of NMDAR1, GAT 1 and GAT 3 was increased, relatively to normal group. Interestingly, BBPs at all the tested concentrations significantly increased the GAD 65 (*P* < 0.05, *P* < 0.01, *P* < 0.01). BBPs significantly increased the protein expressions of GABAA-Rα1 (*P* < 0.05, *P* < 0.01) and decreased GAT 1 (*P* < 0.01, *P* < 0.01) and GAT 3 (*P* < 0.05, *P* < 0.01) at the concentrations of 400 and 800 μg/mL. Only BBPs at a concentration of 800 μg/mL obviously increased GAD 67 (*P* < 0.05) expression and decreased NMDAR1 (*P* < 0.01) expression in PC12 cells injured by Glu, relative to the model cells.

## 3. Discussion

The present study was aimed at investigating the neuroprotective effect of protein extracts derived from *Bombyx batryticatus* in an in vitro model of epilepsy. The effect of BBPs on cell viabilities of PC12 cells injured by Glu was detected in Figure 1, and results showed that BBPs could decrease the Glu-toxic effects, and presents certain neuroprotective effect.

The occurrence of epilepsy is related to the content imbalance of GABA (inhibitory of neurotransmission) and Glu (excitatory neurotransmitter) [5]. Contents of neurotransmitter (GABA and 5-HT), cytokines (IL-1β, TNF-α and IL-4) and GR were detected in the present study. Results in Figure 2 showed that BBPs decreased contents of IL-1β, IL-4 and TNF-α in NGF-induced PC12 cells injured by Glu, whereas up regulated contents of 5-HT and GABA, there were no significant changes of GRα. Above results showed that BBPs decreased the toxic effects caused by Glu treatment, which was related to inhibiting immune response.

Ca^2+^ is the ubiquitous messenger in cells and plays a key role in neuronal signaling and fusion of synaptic vesicles [42]. It is reported that Ca^2+^ is also associated with epilepsy. When the concentration of extracellular Glu increase, excessive excitation of Glu receptor on cell membrane leads to a large influx of Ca^2+^, which increase the concentration of intracellular Ca^2+^. The role of Ca^2+^ dependence may be related to the release of Glu and GABA through voltage-gated channel dysfunction [43]. In the present study, it was found in Figure 3 that the BBPs significantly reduced Ca^2+^ levels in NGF-induced PC12 cells injured by Glu, which demonstrated that BBPs can reduce the toxicity of Glu by regulating intracellular Ca^2+^ concentration.

The above study results demonstrated that BBPs has significant protective effect on NGF-induced PC12 cells injured by Glu. To explore the underlying mechanism, expressions of protein and mRNA related to GABA signaling pathway, including GABAA-Rα1, GAD 65, GAD 67, GAT 1 and GAT 3 were detected, and results were shown in Figure 4 and Figure 5. In addition, protein and mRNA expression of excitatory receptor (NMDAR1) was also investigated in this study. Results in the present study showed that BBPs significantly up-regulated mRNA and protein expressions of GABAA-Rα1, GAD 65 and GAD 67, whereas down-regulated expressions of GAT 1, GAT 3 and NMDAR1. The above results showed that BBPs present protective effects on NGF-induced PC12 cells injured by Glu through regulating GABA signaling pathways.

**Figure 6:**
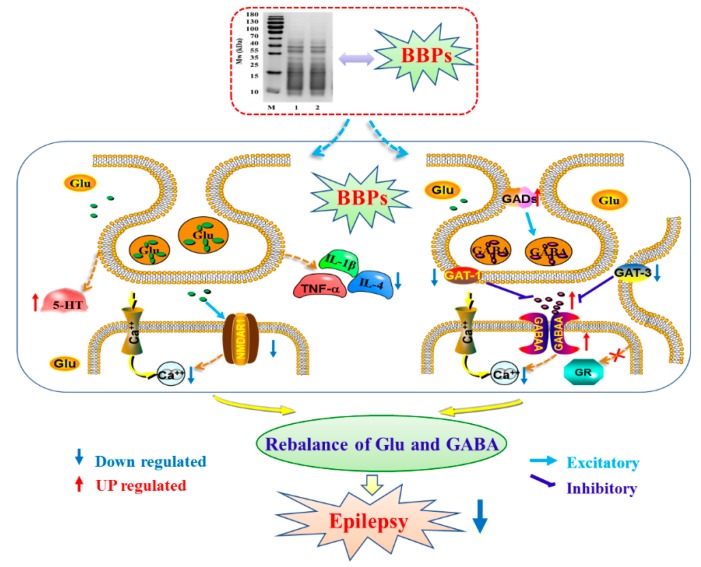
Molecular mechanism of BBPs on epilepsy. BBPs: protein extracts in *Bombyx batryticatus*.

Collectively, the present investigation demonstrated that BBPs presented significantly protective effect on PC12 cells injured by Glu via regulating GABA signaling pathways, inhibiting inflammation and regulating intracellular Ca^2+^ concentration to rebalance Glu and GABA levels between neurons. The possible pathway of protective effect of BBPs on PC12 cells injured by Glu can be briefly shown in Figure 6.

## 4. Materials and Methods

### 4.1. Materials and Chemicals

BB medicinal materials were purchased from Chengdu Min-Jiang-Yuan Pharmaceutical Co. Ltd. (Chengdu, China) and were identified by Prof. Chun-Jie Wu (School of Pharmacy, Chengdu University of Traditional Chinese Medicine, Cheng Du, China). A voucher specimen (Y170628) was deposited in the School of Pharmacy, Chengdu University of Traditional Chinese Medicine (Chengdu, China). Glu were purchased from Sigma-Aldrich Co. (St. Louis, MO, USA). Fetal bovine serum (FBS), and donor equine serum were obtained from Hyclone (collected and Processed in logan, UT, USA). The RPMI-1640 media, phosphate buffer saline (PBS) and 0.25% trypsin-EDTA (1×) (obtained from gibco company, Ontario, made in Canada). Dimethyl Sulfoxide (DMSO) was purchased from Boster Biological Technology Co. Ltd (California, CA, USA). The cell counting kit-8 (CCK-8) was purchased from 4A Biotech Co. Ltd. (Beijing, China). GABA, 5-HT and GRα ELISA Kits were products of Elabscience; IL-1β, IL-4 and TNF-α ELISA kits were products of Multi sciences (lianke) biotech, Co. Ltd. (Hangzhou, China). GABAA-Rα1, NMDAR1, GAD 65, GAD 67, GAT 1 and GAT 3 antibodies were purchased from Abcam (Cambridge, MA, USA). Bicinchoninic acid (BCA) protein assay reagent and horseradish peroxidase- (HPR-)conjugated secondary antibody were purchased from Beyotime Institute of Biotechnology (Shanghai, China). RNA TRIzol® Reagent was purchased from Ambion (Shanghai, China). PrimeScript RT reagent Kit and TB Green^TM^ Premix Ex Taq^TM^ II (Tli RNaseH Plus) were purchase from Takara Bio (Bejing, China). All other reagents used in the experiments were of analytical grade.

### 4.2. Extraction of BBPs by Ultrasonic-Assisted Extraction (UAE)

Minimal degradation of the protein amino acid backbone or dephosphorylation is essential to preserve the analytical utility of the extract [44]. In the present study, UAE was employed for the extraction of BBPs. BB were powdered defatted with petroleum ether (1:5, *w*/*v*). Briefly, 10 g sample was extracted by phosphate buffer (pH 8.0, 30 mM) using ultrasonic cleaning machine (Tianjin Auto science Instrument Co., Ltd., Tianjin, China). The following extraction conditions were used: ultrasonic power (140, 180, 220, 260, and 300 W), extraction time (0.25, 0.5, 0.75, 1 and 1.25 h) and ratio of phosphate buffer to raw material 2:1, 3:1, 4:1, 5:1 6:1 (*v*/*w*). After extraction, the solutions were collected and centrifuged (10,000 rpm, 30 min). The soluble proteins in the supernatant was fractional precipitated by saturated (80%) ammonium sulfate ((NH4)_2_SO_4_) at 4 °C overnight and then centrifuged at 5000 rpm for 30 min. The obtained precipitates were dissolved in PBS and dialyzed at 4 °C for 24 h against distilled water using 30 kDa dialysis membranes and lyophilized [45]. The protein content was determined by BCA method.

Yield (%) = W_1_/W_0_ × 100 (1)

The W_1_ for protein content (g), W_0_ for dry defatted powder of *Bombyx Batryticatus* (g).

To obtain an optimal extraction of BBPs, response surface methodology (RSM) based on Box-Benhnken design (BBD) was carried out (Table 2). These three factors of extraction time (0.75, 1 and 1.25 h), ratio of water to raw material (3, 4 and 5 mL/g) and ultrasonic power (180, 220 and 260 W) were designated as A, B and C, respectively. Seventeen experiments based on BBD with three center points were performed in random order.

All the tests were repeated for three times, and the analysis of variance (ANOVA) was conducted to analysis the BBD results. Design Expert (Version 8.0.6, Stat-Ease, Minneapolis, MN, USA) software was used to estimate the response of independent variables. Response surfaces were drawn to determine the individual and interactive effects of test variable on response. Additional confirmation experiments were subsequently conducted to verify the validity of the statistical experimental design.

### 4.3. Cell Culture

Rat pheochromocytoma-derived cell line PC12 cells were obtained from Wuhan Pu-nuo-sai Life Technology Co. Ltd. (Wuhan, China) and maintained in RPMI-1640 medium supplemented with 5% horse serum, 5% fetal bovine serum (FBS) and 1% penicillin/streptomycin. Cells were incubated at 37 °C in a 5% CO_2_ atmosphere. For cell differentiation, cells were treated with 50 ng/mL of nerve growth factor (NGF; Sigma-Aldrich, St. Louis, MO., USA) for 48 h [46]. NGF-induced cells without BBPs and Glu were normal groups; NGF-induced cells without BBPs, but with 20 mmol/L Glu were model groups.

### 4.4. Cell Viability Assay

The cell viability was evaluated by the CCK-8 (Cell Counting Kit-8) assay. Cells (30,000 cells/well) were inoculated in 96-well plates for 24 h. To evaluate the effect of BBPs on cell viability, BBPs at the final concentrations of 50, 100, 200, 400, and 800 μg/mL were added and cultured at 37 °C for 24 h 5% CO_2_. Afterwards, 10 μL of CCK-8 solution were added to each well. The 96-well plates were maintained at 37 °C for 1 h and the absorbance was measured using microplate reader (Bio-rad; imark) at 450 nm. All groups were repeated in triplicate.

To evaluate the effect of Glu on the NGF-induced PC12 cells viabilities, cells were presented with Glu at concentrations of 5, 10, 20, 40 and 80 mmol/L for 24, 48 and 72 h at 37 °C. In addition, to evaluate the effect of BBPs on NGF-induced PC12 cells of Glu-damaged viabilities, BBPs at concentrations of 50, 100, 200, 400 and 800 μg/mL for 24 h at 37 °C and subsequently subjected to Glu at the concentration of 20 mmol/L for 24 h. CCK-8 assay was performed as the above method.

### 4.5. Determination of GABA, IL-1β, IL-4, TNF-α and GRα Content in PC12 Cells

Cells were inoculated into the 24-well plates for 24 h. BBPs at the final concentrations of 200, 400, and 800 μg/mL were added and cultured for another 24 h. Next, Glu at the final concentration of 20 mmol/L was added and cultured for a further 24 h. Then, media were collected, centrifuged to eliminate cell debris, and stored at 80 °C until use. Contents of GABA, IL-1β, IL-4, TNF-α and GRα were determined by ELSA kits following the manufacturer’s instruction using a Multlskan Mk3 Microplate Reader (Thermo Fisher, Waltham, MA, USA).

### 4.6. Measurement of Cytosolic Free Calcium (Ca^2+^) Release

To study molecular mechanisms of protective effects of BBPs on NGF-induced PC12 cells injured by Glu. Further, Ca^2+^ levels in cells were detected according to the method reported [47]. Cells were inoculated into the 24-well plates for 24 h. BBPs at the final concentrations of 200, 400 and 800 μg/mL were added and cultured for another 24 h. Next, Glu at the final concentration of 20 mmol/L was added and cultured for 24 h. Cells were harvested, washed using PBS and stained by Fluo-3 AM kit, then incubated for 20 min at 37 °C. The levels of Ca^2+^ were detected by flow cytometry assay on a FACS Calibur flow cytometer (BD Biosciences, San Diego, CA, USA). All experiments were repeated thrice.

### 4.7. Quantitative Real-Time Polymerase Chain Reaction (RT-PCR) Assay

Total RNA of the NGF-induced PC12 cells was extracted according to the manufacturer’s instruction, and their purity and concentration were determined by their absorbance at 260 and 280 nm. Then, 2 μg RNA was reversely transcribed into cDNA using the Revert Aid First Strand cDNA Synthesis Kit. RT-PCR was performed using an ABI Step OnePlus System (Applied Bio systems, Foster City, CA, USA). The reaction process for the RT-PCR was as follows: 95 °C for 30 s, 95 °C for 5 s, 55 °C for 30 s, and 72 °C for 30 s looping 45 times and sequence of primers was showed in Table 3. The gene expressions of GABAA-Rα1, GAD 65, GAD 67, GAT 1 NMDAR1 and GAT 3 were normalized to *β*-actin and analyzed by using 2^−^^△△CT^ method.

### 4.8. Western Blot Assay

Total proteins of the NGF-induced PC12 cells were extracted, and protein concentration was determined using BCA protein assay reagent. Total proteins (35 μg) were separated by 10% SDS-PAGE and 6% SDS-PAGE, then transferred onto a PVDF membrane. After that, the PVDF membrane was incubated with primary antibodies of GABAA-Rα1 (1:1000), NMDAR1 (1:1000), GAD 65 (1:1000), GAD 67 (1:1000), GAT-1 (1:1000) and GAT-3 (1:1000) at 4 °C overnight. The membrane was washed and further incubated with HPR-conjugated secondary antibodies (1:5000) at room temperature for 1 h. Protein bands were detected by chemiScope series (Clinx Science Instruments Co., Ltd., shanghai, China) and were evaluated quantitatively, *β*-actin (1:2500) was used as the internal reference. All experiments were repeated thrice.

### 4.9. Statistical Analysis

Data are presented as mean ± standard deviations (SD). Statistical comparisons were made by Student’s t-test or one-way analysis of variance (ANOVA) using GraphPad Prism 5 software (GraphPad Software Inc., La Jolla, CA, USA), followed by Fisher least-significant difference to test the means that were significantly different from the control means. *P* < 0.05 was set as the significant level.

## 5. Conclusions

In conclusion, all of the findings of the present study supported the evidence for the neuroprotective effect of protein extracts obtained from *Bombyx batryticatus* on NGF-induced PC12 cells injured by Glu. The underlying molecular mechanism might regulate the GABA signaling pathway. Results of in the present investigation may provide certain scientific basis for the clinical application of *Bombyx batryticatus* for the treatment of epilepsy.

## Figures and Tables

**Table 1:** ANOVA for the response surface quadratic model.

Source	Sum of Squares	df	Mean Square	*F* Value	*p*-Value (Prob > *F*)
Model	2.59	9	0.29	151.46	<0.0001
A-A	0.039	1	0.039	20.59	0.0027
B-B	0.070	1	0.070	36.94	0.0005
C-C	0.58	1	0.58	303.54	<0.0001
AB	0.14	1	0.14	75.86	<0.0001
AC	4 × 10^−4^	1	4 × 10^−4^	0.21	0.6606
BC	4.225 × 10^−3^	1	4.225 × 10^−3^	2.22	0.1799
A2	0.64	1	0.64	334.28	<0.0001
B2	0.35	1	0.35	181.24	<0.0001
C2	0.60	1	0.60	313.13	<0.0001
Residual	0.013	7	1.904 × 10^−3^		
Lack of Fit	6.325 × 10^−3^	3	2.108 × 10^−3^	1.20	0.4153
Pure Error	7 × 10^−3^	4	1.750 × 10^−3^	--	--
Cor Total	2.61	16		--	--
Standard deviation	0.044	R^2^	0.9949	--	--
C.V.%	2.19	Adj R^2^	0.9883	--	--
Adeq Precision	32.686	Pred R^2^	0.9570	--	--

**A**: extraction time; **B**: ratio of water to raw material **C**: ultrasonic power.

**Table 2:** Box–Behnken experimental design and results for extraction yield.

Run	Extracting Time (A) (h)	Material Liquid Than (B) (mL/g)	Ultrasonic Power (C) (W)	Extraction Yield (%)
1	0.75	3.00	220	1.64
2	1.25	3.00	220	2.18
3	0.75	5.00	220	1.83
4	1.25	5.00	220	1.61
5	0.75	4.00	180	1.38
6	1.25	4.00	180	1.48
7	0.75	4.00	260	1.95
8	1.25	4.00	260	2.09
9	1.00	3.00	180	1.71
10	1.00	5.00	180	1.46
11	1.00	3.00	260	2.13
12	1.00	5.00	260	2.01
13	1.00	4.00	220	2.48
14	1.00	4.00	220	2.44
15	1.00	4.00	220	2.51
16	1.00	4.00	220	2.47
17	1.00	4.00	220	2.55

**Table 3:** Sequence of primers used for RT-PCR.

Gene Name	Forward Primer	Reverse Primer	Product Length
**β-actin**	*GAAGATCAAGATCATTGCTCC*	*TACTCCTGCTTGCTGATCCA*	111 bp
**GABAA-Rα1**	*CGCTCAGTGGTTGTGGCAGAAGATGG*	*GTCACGGTCAGAACGGTCGTCACTCC*	272 bp
**GAD 67**	*GTGCTGCTCCAGTGTTCTGCCATCC*	*AATCCCACAGTGCCCTTTGCTTTCCA*	203 bp
**GAD 65**	*CAAGTGGAAGCTGAACGGTGTGGAGA*	*TCTGACCAGGAGAGCCGAACATTGC*	100 bp
**NMDAR1**	*GGCACACAGGAGCGGGTAAACAACAG*	*AAGCGGTCCAGCAGGTACAGCATCA*	296 bp
**GAT 1**	*TTGGCTGGCGGGCGTGTTTCTCTTCA*	*TGCGGCTGCTCAGGACCATTCTCA*	246 bp
**GAT 3**	*GGTGCTGGCTCATGGCTCTGTCCT*	*AAGTGCGTCTCCTTCTCTGTGATGGC*	238 bp

## References

[1] Gooneratne I.K., Green A.L., Dugan P., Sen A., Franzini A., Aziz T., Cheeran B. (2016). Comparing neurostimulation technologies in refractory focal-onset epilepsy. J. Neurol. Neurosurg. Psychiatry.

[2] Weber Y.G., Biskup S., Helbig K.L., Von Spiczak S., Lerche H. (2017). The role of genetic testing in epilepsy diagnosis and management. Expert Rev. Mol. Diagn..

[3] Mula M. (2015). Investigational new drugs for focal epilepsy. Expert Opin. Investig. Drugs.

[4] Manford M. (2017). Recent advances in epilepsy. J. Neurol..

[5] Guo X., Yongyan W.U., Song D., Yan Z., Liu T. (2014). Compounds isolated and purified from chloroform active part of bombyx batryticatus and their anticonvulsive activities. Chin. J. Pharm..

[6] Karpova M.N., Kuznetzova L.V., Klishina N.Y. (2015). Gaba and its Receptors in Pathogenesis of Epilepsy. Uspekhi fiziologicheskikh nauk.

[7] Qiu W.J., Hu X.W. (2014). Research progress on pathogenesis and treatment of epilepsy. Chin. J. Clin. Phys..

[8] Obata K. (2013). Synaptic inhibition and γ-aminobutyric acid in the mammalian central nervous system. Proc. Jpn. Acad..

[9] Pinal C.S., Tobin A.J. (1998). Uniqueness and redundancy in GABA production. Perspect. Dev. Neurobiol..

[10] Soghomonian J.-J., Martin D.L. (1998). Two isoforms of glutamate decarboxylase: Why?. Trends Pharmacol. Sci..

[11] Stork O., Ji F.-Y., Kaneko K., Stork S., Yoshinobu Y., Moriya T., Shibata S., Obata K. (2000). Postnatal development of a GABA deficit and disturbance of neural functions in mice lacking GAD65. Brain Res..

[12] Dalby N.O. (2003). Inhibition of γ-aminobutyric acid uptake: Anatomy, physiology and effects against epileptic seizures. Eur. J. Pharmacol..

[13] Jing G., Zhu M.D. (2012). Advances in the pathogenesis of epilepsy. J. Shenyang Med. Coll..

[14] Ravizza T., Vezzani A. (2006). Status epilepticus induces time-dependent neuronal and astrocytic expression of interleukin-1 receptor type I in the rat limbic system. Neuroscience.

[15] Dey A., Kang X., Qiu J., Du Y., Jiang J. (2016). Anti-Inflammatory Small Molecules to Treat Seizures and Epilepsy: From Bench to Bedside. Trends Pharmacol. Sci..

[16] Sonar S., Lal G. (2015). Role of Tumor Necrosis Factor Superfamily in Neuroinflammation and Autoimmunity. Front. Immunol..

[17] Zhang X., Li X., Liu N., Zheng P., Ma L., Guo F., Sun T., Zhou R., Yu J. (2019). The Anticonvulsant Effects of Baldrinal on Pilocarpine-Induced convulsion in Adult Male Mice. Molecules.

[18] Shandra A.A., Godlevsky L.S., Vastyanov R.S., Oleinik A.A., Konovalenko V.L., Rapoport E.N., Korobka N.N. (2002). The role of TNF-alpha in amygdala kindled rats. Neurosci. Res..

[19] Li T., Zhai X., Jiang J., Song X., Han W., Ma J., Xie L., Cheng L., Chen H., Jiang L. (2017). Intraperitoneal injection of IL-4/IFN-γ modulates the proportions of microglial phenotypes and improves epilepsy outcomes in a pilocarpine model of acquired epilepsy. Brain Res..

[20] Pijnenburg-Kleizen K.J., Engels M., Mooij C.F., Griffin A., Krone N., Span P.N., Van Herwaarden A.E., Sweep F.C.G.J., Der Grinten H.L.C.-V. (2015). Adrenal Steroid Metabolites Accumulating in Congenital Adrenal Hyperplasia Lead to Transactivation of the Glucocorticoid Receptor. Endocrinology.

[21] Yu C.-W., Chang P.-T., Hsin L.-W., Chern J.-W. (2013). Quinazolin-4-one Derivatives as Selective Histone Deacetylase-6 Inhibitors for the Treatment of Alzheimer’s Disease. J. Med. Chem..

[22] Nishina A., Kimura H., Tsukagoshi H., Kozawa K., Koketsu M., Ninomiya M., Sato D., Obara Y., Furukawa S. (2013). Neurite outgrowth of PC12 cells by 4’-O-β-D-glucopyranosyl-3’,4-dimethoxychalcone from Brassica rapa L. ‘hidabeni’ was enhanced by pretreatment with p38MAPK inhibitor. Neurochem. Res..

[23] Terada K., Matsushima Y., Matsunaga K., Takata J., Karube Y., Ishige A. (2018). The Kampo medicine Yokukansan (YKS) enhances nerve growth factor (NGF)-induced neurite outgrowth in PC12 cells. Bosn. J. Basic Med. Sci..

[24] Ma K., Yan N., Huang Y., Cao G., Deng J., Deng Y. (2014). Effects of nerve growth factor on nerve regeneration after corneal nerve damage. Int. J. Clin. Exp. Med..

[25] Zeng X., Hu K., Chen L., Zhou L., Luo W., Li C., Zong W., Chen S., Gao Q., Zeng G. (2018). The effects of ginsenoside compound k against epilepsy by enhancing the gamma-aminobutyric acid signaling pathway. Front. Pharmacol..

[26] Kim M.H., Lee H.J., Lee S.-R., Lee H.-S., Huh J.-W., Bae Y.C., Lee D.-S. (2019). Peroxiredoxin 5 Inhibits Glutamate-Induced Neuronal Cell Death through the Regulation of Calcineurin-Dependent Mitochondrial Dynamics in HT22 Cells. Mol. Cell. Biol..

[27] Soukupová M., Binaschi A., Falcicchia C., Palma E., Roncon P., Zucchini S., Simonato M. (2015). Increased extracellular levels of glutamate in the hippocampus of chronically epileptic rats. Neuroscience.

[28] Albrecht J., Zielińska M. (2017). Mechanisms of Excessive Extracellular Glutamate Accumulation in Temporal Lobe Epilepsy. Neurochem. Res..

[29] Yu S.P., Strasser U., Tian M., Choi D.W.J.S. (1999). Nmda receptor-mediated k+ efflux and neuronal apoptosis. Science.

[30] Zhang J., An S., Hu W., Teng M., Wang X., Qu Y., Liu Y., Yuan Y., Wang D. (2016). The Neuroprotective Properties of Hericium erinaceus in Glutamate-Damaged Differentiated PC12 Cells and an Alzheimer’s Disease Mouse Model. Int. J. Mol. Sci..

[31] Huang H., Peng X., Peng Y. (2003). Modern research progress of bombyx mori. J. Hunan. Univ. Tradit. Chin. Med..

[32] Wu J.-Y., Sheikho A., Ma H., Li T.-C., Zhao Y.-Q., Zhang Y.-L., Wang D. (2017). Molecular mechanisms of Bombyx batryticatus ethanol extract inducing gastric cancer SGC-7901 cells apoptosis. Cytotechnology.

[33] Zhao Q., Jia T.Z., Cao Q.C., Tian F., Ying W.T. (2018). A Crude 1-DNJ Extract from Home Made Bombyx Batryticatus Inhibits Diabetic Cardiomyopathy-Associated Fibrosis in db/db Mice and Reduces Protein N-Glycosylation Levels. Int. J. Mol. Sci..

[34] Yan H., Wang G.J., Wang J., Chu Y.L. (2004). Advances in studies on constituents and pharmacological actions of bombyx mori. China. Seri. Cul..

[35] Hu M., Yu Z., Wang J., Fan W., Liu Y., Li J., Xiao H., Li Y., Peng W., Wu C. (2017). Traditional Uses, Origins, Chemistry and Pharmacology of Bombyx batryticatus: A Review. Molecules.

[36] Hu M., Liu Y., He L., Yuan X., Peng W., Wu C. (2019). Antiepileptic Effects of Protein-Rich Extract from Bombyx batryticatus on Mice and Its Protective Effects against H2O2-Induced Oxidative Damage in PC12 Cells via Regulating PI3K/Akt Signaling Pathways. Oxid. Med. Cell. Longev..

[37] Koo B.-S., An H.-G., Moon S.-K., Lee Y.-C., Kim H.-M., Ko J.-H., Kim C.-H. (2003). Bombycis corpus extract (BCE) protects hippocampal neurons against excitatory amino acid-induced neurotoxicity. Immunopharmacol. Immunotoxicol..

[38] Bai Y., Zhao Q., He M., Ye X., Zhang X. (2019). Extensive characterization and differential analysis of endogenous peptides from Bombyx batryticatus using mass spectrometric approach. J. Pharm. Biomed. Anal..

[39] Cheng S.-M., Huang J., Wang H.-Y., Li G.-Y., Lin R.-C., Wang J.-H. (2014). Two new compounds from Bombyx batryticatus. J. Asian Nat. Prod. Res..

[40] Shindyapina A.V., Komarova T.V., Sheshukova E.V., Ershova N.M., Tashlitsky V.N., Kurkin A.V., Yusupov I.R., Mkrtchyan G.V., Shagidulin M.Y., Dorokhov Y.L. (2017). The Antioxidant Cofactor Alpha-Lipoic Acid May Control Endogenous Formaldehyde Metabolism in Mammals. Front. Neurosci..

[41] Caruso G., Fresta C.G., Martinez-Becerra F., Antonio L., Johnson R.T., De Campos R.P.S., Siegel J.M., Wijesinghe M.B., Lazzarino G., Lunte S.M. (2017). Carnosine modulates nitric oxide in stimulated murine RAW 264.7 macrophages. Mol. Cell. Biochem..

[42] Magarkar A., Jurkiewicz P., Allolio C., Hof M., Jungwirth P. (2017). Increased Binding of Calcium Ions at Positively Curved Phospholipid Membranes. J. Phys. Chem. Lett..

[43] Krisanova N., Pozdnyakova N., Pastukhov A., Dudarenko M., Maksymchuk O., Parkhomets P., Sivko R., Borisova T. (2019). Vitamin D3 deficiency in puberty rats causes presynaptic malfunctioning through alterations in exocytotic release and uptake of glutamate/GABA and expression of EAAC-1/GAT-3 transporters. Food Chem. Toxicol..

[44] Ericsson C., Nistér M. (2011). Protein extraction from solid tissue. Methods. Mol. Biol..

[45] Chen Y., Li C., Zhu J., Xie W., Hu X., Song L., Zi J., Yu R. (2017). Purification and characterization of an antibacterial and anti-inflammatory polypeptide from Arca subcrenata. Int. J. Biol. Macromol..

[46] Lin K.H., Li C.Y., Hsu Y.M., Tsai F.J., Tang C.H., Yang J.-S., Wang Z.H., Yin M.C. (2019). Oridonin, A natural diterpenoid, protected NGF-differentiated PC12 cells against MPP+- and kainic acid-induced injury. Food Chem. Toxicol..

[47] Overbeeke R., Yildirim M., Reutelingsperger C.P., Haanen C., Vermes I. (1999). Sequential occurrence of mitochondrial and plasma membrane alterations, fluctuations in cellular Ca2+ and pH during initial and later phases of cell death. Apoptosis.

